# A Case Report of Pulmonary Exacerbation after Initiation of Lumacaftor/Ivacaftor Therapy in a CF Female with Complicated Lung Disease

**DOI:** 10.1155/2018/8394370

**Published:** 2018-05-30

**Authors:** Elpis Hatziagorou, Eleana Kouroukli, Vasiliki Georgopoulou, John Tsanakas

**Affiliations:** ^1^3rd Paediatric Department, CF Unit, Hippokration General Hospital of Thessaloniki, Aristotle University of Thessaloniki, Greece; ^2^Radiology Department, Hippokration General Hospital of Thessaloniki, Greece

## Abstract

Novel targeted treatments for Cystic Fibrosis give rise to new hope for an ever-growing number of CF patients with various mutations. However, very little evidence and guidelines exist to steer clinical decisions regarding patients whose illness takes an unexpected course. In such cases, the benefits and risks of discontinuing these treatments must be carefully and individually weighed, since their long-term effects remain mainly uncharted territory. In this report we document the case of a homozygous F508del CF patient with severe lung disease who presented with a pulmonary exacerbation shortly after the beginning of treatment with lumacaftor/ivacaftor and the complicated initial phase of therapy, which was followed by significant improvements.

## 1. Introduction

In the few years since their FDA approval and introduction into Cystic Fibrosis (CF) care and management, cystic fibrosis transmembrane conductance regulator (CFTR) modulators are changing the face of CF care with demonstrated benefits in various domains, even bearing the promise to modify disease progression [1]. The combination lumacaftor/ivacaftor (LUM/IVA) was the first of these drugs to receive FDA approval for the treatment of patients homozygous for the Phe508del CFTR mutation. Lumacaftor increases the amount of mutated CFTR protein reaching the cell surface, while ivacaftor enhances the open probability of rescued CFTR channels already on the cell surface. Patients with the Phe508del mutation benefit from the combined action of the two agents, which results in an increased amount of chloride being transferred through the cell membrane [2]. Commonest side-effects comprise respiratory and gastrointestinal symptoms, along with elevated liver enzymes, but overall the drug demonstrated a safe profile and was generally well-tolerated in phase 3 trials [1].

Herein we document the case of a patient who was prescribed LUM/IVA and the therapeutic dilemma that occurred shortly after treatment initiation. The patient provided written informed consent for the publication of this report.

## 2. Case Report

The patient is a 22-year-old woman of Caucasian origin diagnosed with CF shortly after birth, due to meconium ileus, which was operated on the second day of life. Diagnosis was confirmed with sweat testing and DNA mutation analysis which revealed F508del homozygosity. At nine years she underwent further abdominal surgery for DIOS and at 13 years she presented with nasal polyps, which were treated surgically, too. She has been colonized with* Pseudomonas aeruginosa* (PsA) since the age of 8 years and subsequently she has been receiving an average of two courses of i.v. antipseudomonal antibiotics per year.

At the age of 14 years her FEV1 was 95% predicted and her BMI 26.4 kg/m^2^. The same year she was diagnosed with allergic bronchopulmonary aspergillosis (ABPA). She was started on oral corticosteroids and itraconazole and was complicated with invasive aspergillosis that was successfully treated with i.v. Liposomal Amphotericin. Eventually the patient was discharged home on oral Voriconazole and Prednisolone. After the initiation of steroids, the patient also developed diabetes, which was treated with subcutaneous insulin. An HRCT scan obtained a month later showed marked improvement of the lung disease and a course of anti-IgE monoclonal antibody (Omalizumab) was initiated and continued for 13 months.

Following her complete recovery from ABPA, she remained stable, with her prior respiratory symptoms in remission, but her FEV_1_ had dropped to 75%. She was receiving insulin, Pulmozyme, inhaled antibiotics, and 2 courses of i.v. antibiotics per year for her PsA chronic infection. Shortly after omalizumab discontinuation, however, she suffered a severe pulmonary exacerbation, following which her FEV_1_ dropped abruptly to 48%. After two weeks of iv antipseudomonal antibiotics and intensive pulmonary rehabilitation in the hospital, her FEV_1_ rose to almost 60%, but never reached her previous levels again.

In the years that followed, she continued to experience 2-3 exacerbations per year, which were treated with iv antipseudomonal antibiotics. She was receiving inhaled tobramycin and her FEV_1_ levels ranged steadily between 50 and 60%. Her liver function tests remained within normal range and annual abdominal ultrasounds revealed no discernible focal lesions in the liver and no intra- or extrahepatic biliary dilatation. Two years before LUM/IVA initiation (19 years old), her inhaled tobramycin was switched to aztreonam, and she experienced a substantial improvement in self-reported symptoms and pulmonary function, with FEV_1_ reaching 68% for a brief period. However, at 20 years she suffered another severe exacerbation, with her FEV_1_ dropping to 40%, and saw no significant improvement after iv antibiotics and pulmonary rehabilitation.

At 21 years of age the patient was stable, although with a markedly reduced FEV_1_ of 43.6% predicted, a lung clearance index (LCI) of 24.43, SaO2 97%, a BMI of 19.96 kg/m^2^, and an HbA1c of 6.4%, with three courses of iv antibiotics per year and Azithromycin added to her chronic treatment. In the same year she was started on LUM/IVA therapy. Two weeks after the initiation of LUM/IVA treatment however, she presented with cough and increased amounts of sputum. On chest auscultation there were crackles bilaterally, her oxygen saturation was 92% in room air, and her FEV_1_ was significantly reduced (37%), while her LCI was elevated (28.67). She was eventually hospitalized, after her symptoms further deteriorated and her FEV_1_ dropped to 27%. She was treated with iv antipseudomonal antibiotics and intensive physiotherapy. The pulmonary exacerbation and the significant drop in FEV_1_ after the initiation of LUM/IVA prompted considerations for treatment discontinuation but a decision was made to continue LUM/IVA administration after she started responding to the antibiotics. The next month she presented again with a pulmonary exacerbation (FEV_1_ 34%, SaO_2_ 91%) and received a second course of iv antibiotics, by the end of which her FEV_1_ rose up to 54% and her oxygen saturation was restored to her normal 97%. On her next follow-up visit she appeared significantly improved, in terms of pulmonary function, nourishment, and glycemic control, and even had her insulin doses reduced after reporting that she was experiencing hypoglycemic episodes for the first time. Over the following 12 months of LUM/IVA treatment she experienced sustained improvements in various domains, including absolute change in ppFEV_1_ (ΔFEV1pp=+3.4), LCI (ΔLCI=-9.76), and BMI, kg/m^2^ (ΔBMI=+1). Glycemic control was also improved with reduced need of insulin, while pulmonary exacerbations requiring iv antibiotics were reduced from 3 to one yearly.

## 3. Discussion

Overall, LUM/IVA was proven safe and generally well-tolerated in its phase 3 studies [3], as well as the extension study [1]. Infective pulmonary exacerbations were the most common adverse event and, along with other respiratory events, such as cough, increased sputum, and haemoptysis, they were a common cause of concern and even treatment cessation. However, most of these events occurred at treatment initiation and patients who continued to receive the drug usually saw their symptoms resolve within the first month of treatment [1]. It is proposed that the respiratory events associated with LUM/IVA are caused by an abrupt increase and mobilisation of hypoviscous secretions [4], which often raises concerns about a possible pulmonary exacerbation. However, the exact mechanism is not yet understood and most of these events appear to be transient; therefore, the decision to discontinue treatment on the basis of respiratory events warrants careful assessment.

Accordingly, our patient demonstrated objective signs of a pulmonary exacerbation soon after treatment initiation. These events temporally coincided with the appearance of side-effects in the follow-up studies, and treatment discontinuation was reluctantly considered, due to the short time since initiation. Furthermore, this patient had a history of severe and complicated lung disease and had sustained a significant loss of pulmonary function over the years; therefore, LUM/IVA was expected to provide meaningful benefits. In light of her favorable response to the antibiotics and pulmonary rehabilitation, it was finally decided to continue LUM/IVA administration, which eventually yielded significant improvements. This outcome highlights the fact that the response to these novel agents is individualized and that complicated patients might avoid the respiratory adverse events of LUM/IVA by starting the treatment while being in hospital with close monitoring, intense physiotherapy, and adequate fluid intake.

There are no clear indications as to when treatment with LUM/IVA should be discontinued. In contrast to fixed biomarkers such as liver enzymes, respiratory symptoms are often difficult to objectify, and therefore the decision to stop treatment usually mainly lies in the physician's judgment. It is of utmost importance that every patient's complaint be thoroughly documented and interpreted with caution when initiating a treatment like LUM/IVA, the long-term effects and outcomes of which remain to be explored.
